# Improved fluorescent phytochromes for in situ imaging

**DOI:** 10.1038/s41598-022-09169-x

**Published:** 2022-04-04

**Authors:** Soshichiro Nagano, Maryam Sadeghi, Jens Balke, Moritz Fleck, Nina Heckmann, Georgios Psakis, Ulrike Alexiev

**Affiliations:** 1 Institut Für Pflanzenphysiologie, Justus-Liebig-Universität, 35390 Giessen, Germany; 2 Institut Für Experimentalphysik, Freie Universität Berlin, 14195 Berlin, Germany; 3 Present Address: Faculty of Health Sciences, Department of Food Sciences & Nutrition, Mater Dei Hospital, University of Malta, Msida, MSD 2080 Malta

**Keywords:** Biophysics, Biological fluorescence, Molecular biophysics, Biochemistry

## Abstract

Modern biology investigations on phytochromes as near-infrared fluorescent pigments pave the way for the development of new biosensors, as well as for optogenetics and in vivo imaging tools. Recently, near-infrared fluorescent proteins (NIR-FPs) engineered from biliverdin-binding bacteriophytochromes and cyanobacteriochromes, and from phycocyanobilin-binding cyanobacterial phytochromes have become promising probes for fluorescence microscopy and in vivo imaging. However, current NIR-FPs typically suffer from low fluorescence quantum yields and short fluorescence lifetimes. Here, we applied the rational approach of combining mutations known to enhance fluorescence in the cyanobacterial phytochrome Cph1 to derive a series of highly fluorescent variants with fluorescence quantum yield exceeding 15%. These variants were characterised by biochemical and spectroscopic methods, including time-resolved fluorescence spectroscopy. We show that these new NIR-FPs exhibit high fluorescence quantum yields and long fluorescence lifetimes, contributing to their bright fluorescence, and provide fluorescence lifetime imaging measurements in *E.coli* cells.

## Introduction

**Figure 1:**
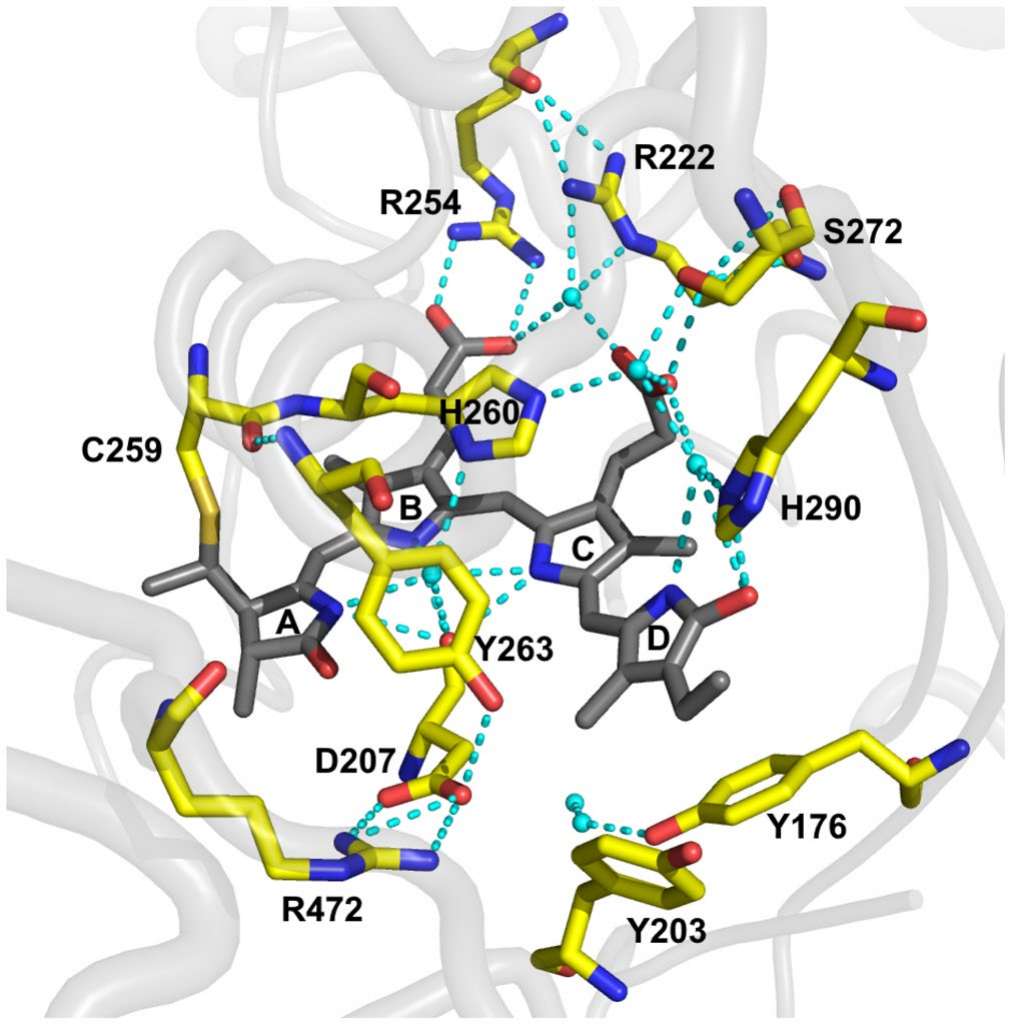
Chromophore binding pocket of Cph1 PGP (pdb 2VEA^7^). The PCB chromophore is covalently attached to C259 and shown in dark grey. Key amino acid residues of the binding pocket are labelled and shown in yellow. Water molecules and hydrogen bonds are shown as cyan spheres and dashed lines, respectively. The figure was created with PyMol^8^.

Phytochromes are natural red/far-red photochromic biliprotein photoreceptors ubiquitous in plants and widespread in prokaryotes and fungi^1^. Phytochromes convert cues from the light environment into biological signals, thus enabling organisms to acclimate^2–4^. Phytochrome photoreceptors use linear tetrapyrrole bilins^5^ such as phytochromobilin (PΦB), phycocyanobilin (PCB), or biliverdin 1Xα (BV) as chromophores, attached to the protein via a thioether bond. Prototypical phytochromes act as photochemical switches that interconvert between stable red (r)—and meta-stable far-red (fr)—absorbing states (Pr and Pfr, respectively). Photoconversion is induced by chromophore isomerisation within the N-terminal photosensory module, which typically comprises PAS, GAF and PHY [Per/ARNT/Sim, cGMP phosphodiesterase/adenyl cyclase/FhlA and phytochrome-specific, respectively] domains—in short PGP—in plant and microbial phytochromes^3^. The *Z* ↔ *E* isomerisation of the linear tetrapyrrole takes place around the C15 = C16 double bond of the methine bridge connecting the pyrrole rings *C* and *D* of the bilin^6^. In plant phytochromes and cyanobacterial phytochromes like *Synechocystis* Cph1, phytochromobilin or phycocyanobilin, respectively, is attached to a conserved cysteine in the GAF domain. A structural model from the chromophore binding pocket of Cph1 is shown in Fig. 1^7^.

Advances in understanding the structure and function of the different domains of phytochromes have allowed phytochrome building blocks to be utilised, amongst others, for the generation of near infrared fluorescent proteins (NIR-FPs) for in vivo imaging^9^. NIR light (~ 650–900 nm) is favourable over shorter wavelengths for biomedical applications as this wavelength range provides an optical window where the absorption by hemoglobin, melanin, and water are low in mammalian tissue. Tissue autofluorescence and also light scattering and absorption by lipids and fat are also low in this range^10^. Thus, the development of fluorescent phytochromes as NIR-FPs is a promising research field for biotechnological and medical applications utilising in vivo imaging^9,11–19^. For instance, several bacteriophytochrome (BphP) -based NIR-FPs were developed as optical tags for use in mammalian systems, including PAiRFP1 and PAiRFP2, IFPs, iRFPs, IFP1.4, or SNFIP^9,11,14,17,18^. However, the fluorescence quantum yield (*Φ*_*f*_) of these NIR-FPs is generally low^11,18^ compared to the fluorescence of phycobiliproteins^19^ and phytofluors^15^ in the orange/red wavelength region. Cyanobacterial phytochromes^12,13^ and cyano-bacteriochromes^16^ are further promising candidates for the design of bright NIR-FPs. Phycobiliprotein subunits might also be useful as NIR-FPs.

Quantum efficiency of chromophore isomerisation and fluorescence quantum yield of photoreceptor proteins seem to be tightly coupled, even though the coupling might be rather complicated^20^. The inhibition of photoconversion by low temperature increases Pr fluorescence, indicating that photoisomerisation provides a major route of energy dissipation^21,22^. Also, inhibition of photoconversion by “locked” chromophores enhanced fluorescence in the bacteriophytochromes Agp1 and Agp2^23^. Similarly, rigidifying the chromophore by attaching the chromophore at two cysteines of the apoprotein increased the fluorescence quantum yield in a BphP iRFP variant^24^. As the protein environment has a critical role in defining the excited state lifetime and thereby the quantum efficiency of bilin photoisomerisation^25^, a higher fluorescence quantum yield in phytochromes may also be achieved by disrupting the photoconversion through appropriate apoprotein mutations^12,13^.

Directed evolution of FPs by random mutagenesis followed by screening for highly fluorescent mutants has been used to identify appropriate mutations. In pioneering work by the Lagarias group, the mutation Y176H in *Synechocystis* Cph1 showed a fluorescence quantum yield of about 14% at the expense of efficient photoconversion to Pfr^12^. Interestingly however, the corresponding substitution failed to enhance fluorescence in bacteriophytochromes^26^. Numerous rounds of mutagenesis using a similar approach lead to a variant of the *Rhodopseudomonas palustris* bacteriophytochrome *Rp*BphP2 with a quantum yield of about 6% after the introduction of no less than 13 mutations onto the PAS-GAF background (iRFP713)^11^. A table with the main characteristics of selected NIR-FPs is provided in the Supporting information (Table S1).

Here we have focused on Cph1 because of the known high fluorescence of Y176H, Y263S and Y263F^13,27^. The 3D structure of the latter has been determined at high resolution^27^. In the present study, we improved the fluorescence of Cph1 variants by the rational approach of combining these mutations. We also studied the effect of PHY domain deletion on fluorescence using the PAS-GAF (PG) construct. In addition, we re-evaluated the exceedingly high fluorescence reported for a mutant of Cph2 from *Synechococcus* OS-B^28^. All the mutants were characterised by steady-state UV–vis absorption, CD and fluorescence spectroscopy. We also provide flash photolysis data. To better understand the fluorescence behaviour of the mutants we measured the fluorescence lifetimes using picosecond time-resolved fluorescence spectroscopy^29–31^. Finally, to translate our results to live imaging, we performed fluorescence lifetime imaging microscopy (FLIM)^31,32^ on *E. coli* cells expressing the highly fluorescent Cph1 PG Y176H/Y263S variant and compared the fluorescence lifetime in *E.coli* to those obtained from the protein in vitro*.*

## Results and discussion

### UV–vis absorption and circular dichroism (CD) spectroscopy

**Figure 2:**
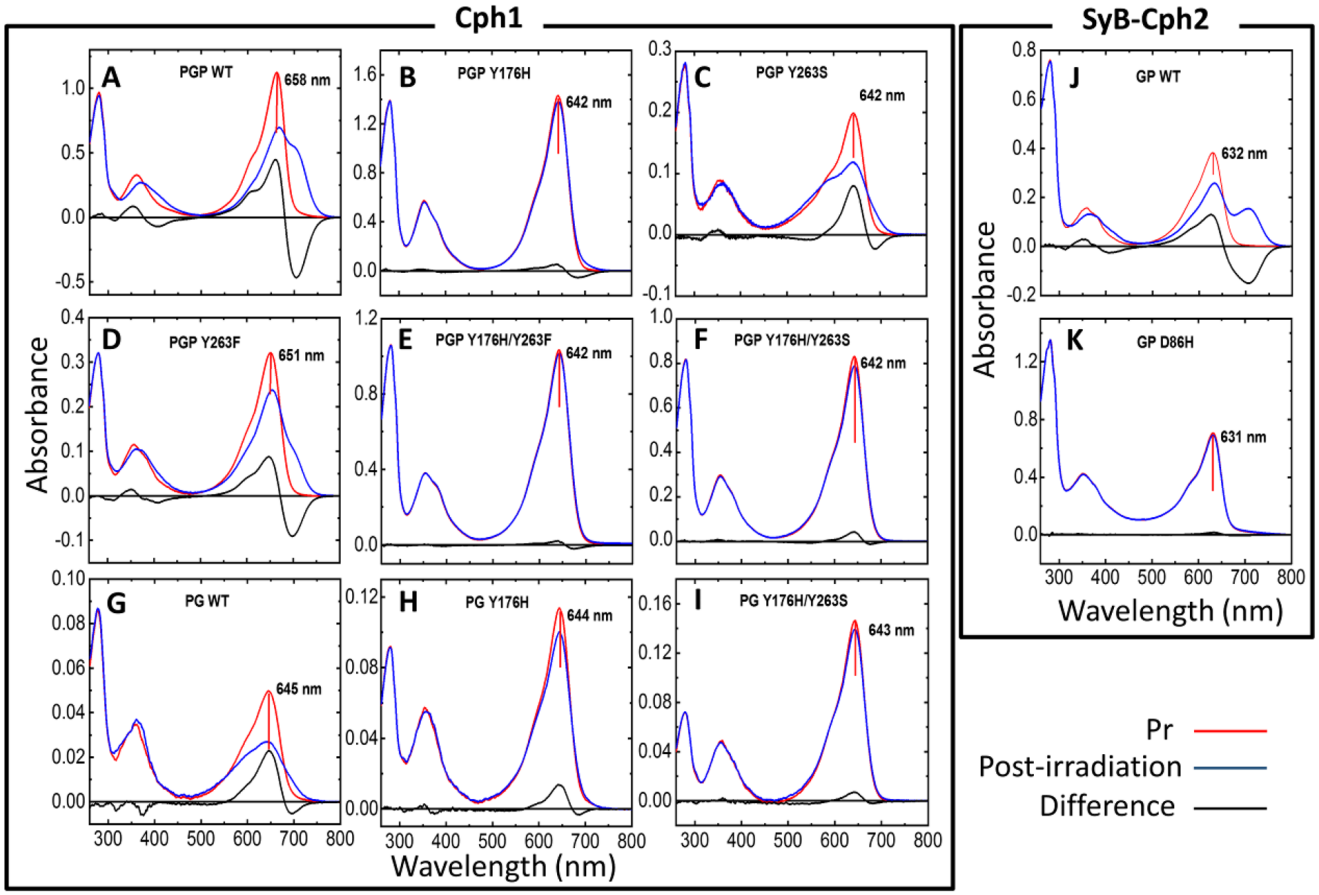
UV–vis absorption spectra of Cph1 PGP, Cph1 PG, SyB-Cph2 GP and variants. The dark-adapted (Pr state) and post-irradiation spectra (red light irradiated sample) are shown in red and blue, respectively. The difference spectra are shown in black. λ_max_ values for Pr are indicated. Cph1 PGP WT and variants are shown in A to F, Cph1 PG WT and variants in G to I. *SyB*-Cph2 WT and its variant are shown in J and K. Buffer conditions: 300 mM NaCl, 50 mM Tris/HCl pH 7.8, 5 mM EDTA, 1 mM β-mercaptoethanol.

Mutant libraries of Cph1 PGP (59 kDa), Cph1 PG (38 kDa), and *SyB*-Cph2 GP (49 kDa) were characterised using UV–vis absorption spectroscopy (Fig. 2). In all variants of Cph1 the Pr absorption peak is blue-shifted compared to the wild type (WT) (Fig. 2, Table 1).

**Table 1:** Spectroscopic properties of Cph1 PGP, Cph1 PG, SyB-Cph2 GP and their variants as shown in Figs. 2, 3 and 4.

Construct	*λ*_*max*_ (ab) (nm)	*λ*_*max*_ (ex) (nm)	*λ*_*max*,_(em) (nm)	Δ*λ*_*max*_, (em–ab) (nm)	Δ*λ*_*max*_*,* (em—ex) (nm)	*Φ*_*f*_	*τ*_mean_ (ns)
Cph1 PGP WT	658	643	675	17	32	0.024	0.83
Cph1 PGP Y176H	642	643	672	30	29	0.144	1.90
Cph1 PGP Y263F	651	652	678	27	26	0.082	1.26
Cph1 PGP Y263S	642	642	669	27	27	0.125	1.46
Cph1 PGP Y176H/Y263S	642	642	670	28	28	0.176	2.00
Cph1 PGP Y176H/Y263F	642	642	672	30	30	0.160	1.92
Cph1 PG WT	645	645	671	26	26	0.018	0.69
Cph1 PG Y176H	644	644	672	28	28	0.108	1.74
Cph1 PG Y176H/Y263S	643	643	669	26	26	0.171	2.00
*SyB* Cph2 GP WT	632	631	660	28	29	0.124	2.07
*SyB* Cph2 GP D86H	631	632	655	24	23	0.202	2.60

Table S2 provides the detailed results for the fluorescence lifetime fits including an exhaustive error analysis. The fluorescence quantum yield *Φ*_f_ is given as fraction.

The Cph1 PGP WT holoprotein undergoes photoconversion from Pr to Pfr, which is associated with the characteristic bathochromic shift of the absorption peak from the red to the far-red region (Fig. 2A). Red light activation of the chromophore leads to isomerisation of the C15 = C16 double bond^13^ and generates the isomerised Lumi-R state^33^. Isomerisation results in the “photoflip” of the *D*-ring, thereby changing the bilin configuration from *ZZZssa* to *ZZEssa*, which is manifested, remarkably, as a hypsochromic shift under denaturing acidic conditions (Fig. S1)^34^. The Y263F mutant behaves analogously, except for a blue-shifted absorption maximum of the Pr band (λ_max Pr_ (ab); − 7 nm compared to WT) and a lower proportion of Pfr at photoequilibrium (Fig. 2D), consistent with a previous report^27^. The acid denaturation assay confirms that, as in the WT, a *15Za* to *15Ea* geometry change takes place in PGP (Y263F after red light irradiation (Fig. S1B). In contrast, the Pr-like absorption peak of Cph1 PGP Y176H (Fig. 2B), which is hypsochromically shifted by 16 nm with respect to WT, hardly changes upon irradiation: Pfr formation is almost undetectable as previously shown^12,13^. The absorption maximum of the Pr-like state under acid-denatured conditions is identical to that of the WT, indicating *15Za* geometry (Fig. S1D). After red light irradiation followed by denaturation the spectra were virtually unchanged, consistent with the loss of photoconversion. Additionally, the Cph1 PGP Y176H CD signal in the red region after red and far-red light irradiation is indistinguishable from that of the WT Pr state (Fig. S2), indicating that the chromophore *D*-ring is still α-facial. The lack of photoconversion in Cph1 PGP Y176H shows the important role of position Y176 in the early Pr to Pfr photoconversion events, in agreement with the pioneering work on this Cph1 variant^12,13^ and work on homologous mutants in plant phytochromes^35^ and bacteriophytochrome from *Deinococcus radiodurans* (*Dr*BphP)^26^. The Y263S mutant shows a similar 16 nm hypsochromic shift of the red band as Y176H (Fig. 2C). Moreover, we used time-resolved absorption spectroscopy (flash photolysis) to follow photoconversion directly (Fig. S3). The absorbance difference transient shows that photoconversion is blocked early on in both Y176H and Y263S mutants.

The double mutant variants Cph1 PGP Y176H/Y263S and Cph1 PGP Y176H/Y263F exhibit the same blue-shifted absorption maximum of the chromophore peak in the Pr state (λ_max Pr_ (ab) = 642 nm) as the single mutant Cph1 PGP Y176H (Fig. 2E,F, Table 1).

For Cph1 PG WT, in which the PHY domain is absent, we observed a hypsochromic shift of 13 nm of the Pr absorption band with respect to its PGP WT counterpart (Fig. 2G, Table 1). The Pr state of Cph1 PG Y176H and PG Y176H/Y263S show similarly blue shifted absorption maxima with λ_max_ values of 644 nm and 643 nm, respectively (Fig. 2H,I, Table 1). With respect to photoconversion, the Cph1 PG Y176H single mutant behaved similarly to the PGP equivalent, although photobleaching of the Pr peak was weaker in the PGP background (3.7% *vs.* 11.9%). Y176H/Y263S mutants behaved similarly to Y176H, and photobleaching in both the PG and PGP backgrounds being about 4.8% (Fig. 2B, F, H, I).

*SyB*-Cph2 PG fragments exhibit a Pr peak λ_max_ _Pr_ (ab) = 632 nm for the WT sample. Photoconversion to Pfr takes place in the WT (Fig. 2J), but not in the D86H mutant (Fig. 2K), in agreement with a previous report^28^.

### Steady-state fluorescence spectroscopy

**Figure 3:**
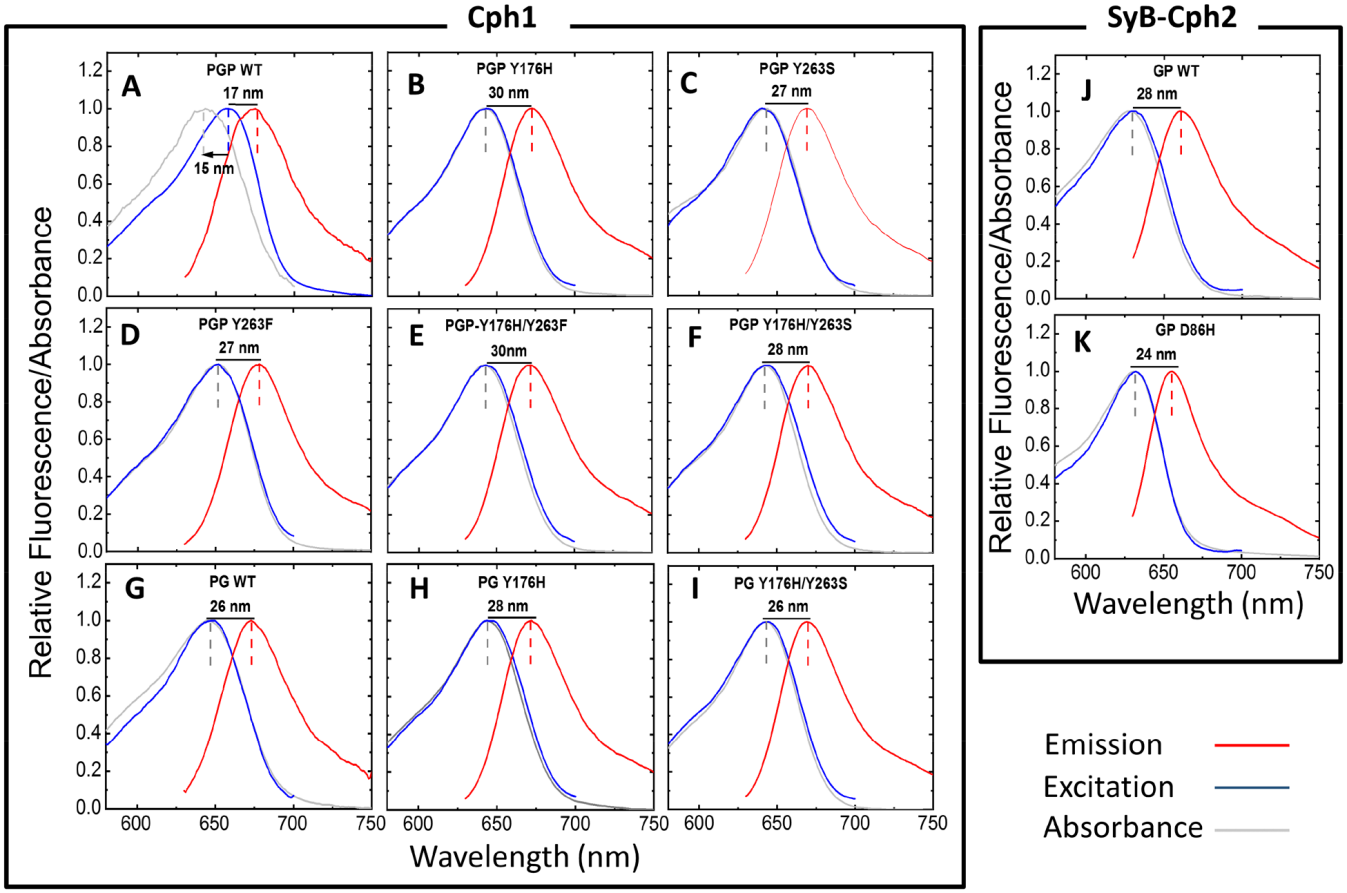
Steady-state absorption, excitation, and emission spectra of Cph1 and SyB-Cph2 variants. The absorption (ab), excitation (ex) and emission (em) spectra are shown in grey, blue, and red, respectively. Cph1 PGP WT and variants are shown in A to F, Cph1 PG WT and variants in G to I, and *SyB*-Cph2 GP WT and its variant in J and K. The Stokes shift (λ_max(em)_ − λ_max(ab)_) is indicated. The blue shift between maximum excitation and absorption wavelength (λ_max(as)_ − λ_max(ex)_) is indicated in A. The values are summarised in Table 1. Conditions as in Fig. 2.

The fluorescence spectroscopic properties of our mutants are shown in Fig. 3 and summarised in Table 1. Notably, the peak of the absorbance spectra and fluorescence excitation spectra of Cph1 PGP WT (658 nm and 643 nm, respectively) do not match, as has been reported earlier^13,27,36^ (and was also observed for Agp1 D197A^23^). This implies the co-existence of substates within Pr and hence the presence of a heterogeneous mixture^36^. It may be hypothesised from the present data that the majority of Cph1 PGP WT represents the substate with longer absorbance wavelength, whereas a minor fraction with a blue-shifted λ_max_(ab) is strongly fluorescent. Indeed, the heterogeneous nature of Pr in Cph1 has been shown by various methods, including absorption spectroscopy^37,38^, magic angle spinning-NMR^39^, polarization-resolved femtosecond visible pump/infrared probe spectroscopy^40^ and dual excitation-wavelength-interleaved transient absorption^41^. Together, these studies provide clear evidence for two (or more) substates of Pr in the electronic ground state, perhaps corresponding to Pr-I and Pr-II previously described^37–41^. The question naturally arises as to which of these corresponds to the fluorescent subspecies. The minor fluorescent fraction might be the Pr-I substate, which showed ~ 35% occupancy based on MAS-NMR data^39^. The idea of conformational heterogeneity may be extended to explain the increased fluorescence of the mutants, whereby the mutations trap the conformation/s exhibiting higher fluorescence. We note that the fluorescence excitation maxima of most Cph1 proteins in this study are similar, implying that the fluorescent substate of the WT and of the fluorescent mutants are structurally related. Further studies are needed here.

The measured fluorescence quantum yield of the Cph1 PGP mutants Y176H (*Φ*_*f*_ = 0.144 ± 0.002) and Y263F (*Φ*_*f*_ = 0.082 ± 0.002) are substantially increased relative to the WT (*Φ*_*f*_ = 0.024 ± 0.001) (Fig. 3A,B,D and Table 1) and in good agreement with earlier measurements^12,27,42^. Y176 and Y263 are conserved among the phytochrome family in plants, cyanobacteria, and bacteriophytochromes. The residue Y176 in strand β1 of the GAF domain of Cph1 (Fig. 1) acts as a molecular gate for the primary *Z*-to-*E* photoisomerisation associated with reorientation of the phenol side chain^12^. This process might be significantly inhibited when tyrosine is replaced with histidine due to its strongly hydrogen-bonded imidazole moiety, perhaps explaining the high fluorescence quantum yield of the Y176H mutant^12^. However attractive this idea seems, it is contradicted by the equivalent mutation in BV-binding bacteriophytochromes not blocking the Pfr formation and not enhancing fluorescence^26^. The origin of this remarkable difference remains to be discovered^43^.

Mutations at position Y263 in Cph1 and equivalent mutations in other phytochromes present a somewhat different picture. Y263 in Cph1 is located in the chromophore binding pocket and interacts with the pyrrole water and the chromophore (Fig. 1). The primary effect of the Y263F mutation is to reduce the quantum efficiency of photoconversion, associated with a corresponding increase in *Φ*_*f*_^27,42^. Minimal structural changes were associated with the mutation, instead an energy-conserving transfer of *D*-ring hydrogen bonding from H290 to Y176 in the WT but missing in Y263F was proposed^42^. By contrast, a recent study showed that the homologous mutation in *Dr*BphP leads to a sheet-to-helix refolding of the tongue connecting the PHY domain with the chromophore binding pocket, the helical conformation resembling that of the WT in the Pfr state^44^. Several water interactions missing in Y263F were suggested to decrease the photoconversion yield^45^. Therefore, the Y263F mutation induces different structural changes in the different phytochromes^27,42,44–46^ and different photophysical behaviour would be expected.

In this report, our primary goal was to test the effect on fluorescence quantum yield by combining mutations known to enhance fluorescence in Cph1. We found a synergistic effect on fluorescence in both double mutants, Cph1 PGP Y176H/Y263F and Cph1 PGP Y176H/Y263S, with fluorescence quantum yields of *Φ*_*f*_ = 0.160 ± 0.002 and *Φ*_*f*_ = 0.176 ± 0.002, respectively, both higher than those of the parental single mutants. Considering our discussion above, the Y176H, Y263F or Y263S single mutants may increase fluorescence by affecting the photoreaction at the preLumi-R step. The higher fluorescence of the Y176H/Y263S double mutant compared to their parental single mutants indicates that there is further potential to increase the fluorescent pathway at the expense of energy dissipation via photoconversion and/or heat. Thus, based on this example we hope that further rational modifications may result in even more efficient NIR-FPs.

We also investigated Cph1 PG variants in which the PHY domain is missing to test the effect of the PHY domain on fluorescence. Cph1 WT, Y176H and Y176H/Y263S as PG constructs show *Φ*_*f*_ values of 0.018 ± 0.001, 0.108 ± 0.002 and 0.171 ± 0.003, respectively, lower than for the respective PGP constructs (Table 1). Removal of the PHY domain from the PGP fragments in *Dr*BphP and *Rp*BphP3, however, increased the *Φ*_*f*_ values from 0.017 to 0.025, and 0.043 to 0.055, respectively^44,47^. Here again, despite the rather similar 3D structures of Cph1 and *Dr*BphP, homologous mutations have quite different effects. Although absence of the PHY domain generally blocks Pfr formation^45,47^, exceptions are *Gm*.phyA^48^, the monomeric mutant of *Dr*BphP^45^ and *Is*PadC^49^ that form Pfr-like states. It might be that the PHY domain tongue contributes to the rigidity of the chromophore pocket, sometimes enhancing and sometimes inhibiting fluorescence, but usually as a requirement for Pfr formation.

As discussed above, increased fluorescence probably results from disruption of the photoconversion pathway at early steps. Despite similar 3D structures, subtle differences in the interactions between the chromophore and its pocket, in particular Y176, Y263 and the tongue (PHY domain) lead to very different effects among the various phytochromes. How these differences arise is currently unclear. We note that the free energy levels of Pr and Pfr are quite similar^21^ and that forward photoreaction from Pr to Pfr is radically different from the Pfr to Pr back reaction. Even small structural differences might have large effects on the relative stabilities of different (sub)states.

### Fluorescence of SyB-Cph2

We were intrigued by the report of Ulijasz et al. (2008) in which a *SyB*-Cph2 GP construct carrying the D86H mutation (equivalent to D207H in Cph1) was said to be "five times more fluorescent" than Cph1 PGP Y176H^28^, corresponding to a fluorescence quantum yield of about 0.6. We prepared holoprotein samples of *SyB*-Cph2 GP WT and D86H, deriving *Φ*_*f*_ = 0.124 ± 0.003 and *Φ*_*f*_ = 0.202 ± 0.003, respectively (Table 1). The difference between the measurement sets might arise from the different excitation wavelengths used to collect the emission spectra, namely 360 nm in the Ulijasz study^28^ vs. 610 nm here and in Maillet et al.^27^ To check this possibility, we measured emission spectra based on both excitation wavelengths, adjusting the dilution appropriately to give similar absorbances at the excitation wavelength. Figure S4 shows that the excitation wavelength used had minimal effect. As we assume that the Soret band around 350 nm corresponds to an S_2_ state that can favourably relax to S_1_, our observation agrees with Kasha’s rule that fluorescence only occurs from the S_1_ excited state to the S_0_ ground state, meaning that fluorescence is independent of excitation wavelength for the same fluorescence state. Ulijasz et al.^28^ standardized the sample concentrations at an absorbance of A_λmax, red_ = 0.6, thereby failing to account for differences in red/Soret band oscillator ratios between different holoproteins when exciting the Soret band at 360 nm. Although this would clearly have led to different excitation and biased quantification, the oscillator ratio of the *SyB*-Cph2 GP D86H construct cannot explain the discrepancy in fluorescence quantum yield. We consider that the differences in fluorescence quantum yield derives from a dilution error resulting in a much higher concentration of that sample than given in the paper, or simply from using a factor for calculation of the standardized concentration without actually diluting the sample. The fluorescence excitation spectrum of *SyB*-Cph2 GP D86H shown by Ulijasz et al.^28^ differs dramatically not only from those of other constructs in that work but also from that of its own absorbance spectrum. While unusual, such differences are perhaps not impossible. However, in our present study the excitation and absorption spectra of *SyB*-Cph2 GP D86H are similar at a low protein concentration (Fig. 3 and Fig. S5), as it would be expected. On the other hand, when we increased the sample concentration to ~ 30 µM (~ 1.5 mg/ml or higher), we were able to reproduce an artefactual excitation spectrum similar to that of Ulijasz et al*.*^28^ (Fig. S5). In fluorescence spectroscopy such artefacts arise typically at high sample concentrations as the result of excitation light gradients and fluorescence reabsorption. Naturally, such errors would have been carried through into the calculation of the fluorescence quantum yield. Thus *SyB*-Cph2 GP D86H exhibits a very high fluorescence quantum yield of about 0.2 (Table 1), not 0.6 as published^28^.

### Time-resolved fluorescence spectroscopy

**Figure 4:**
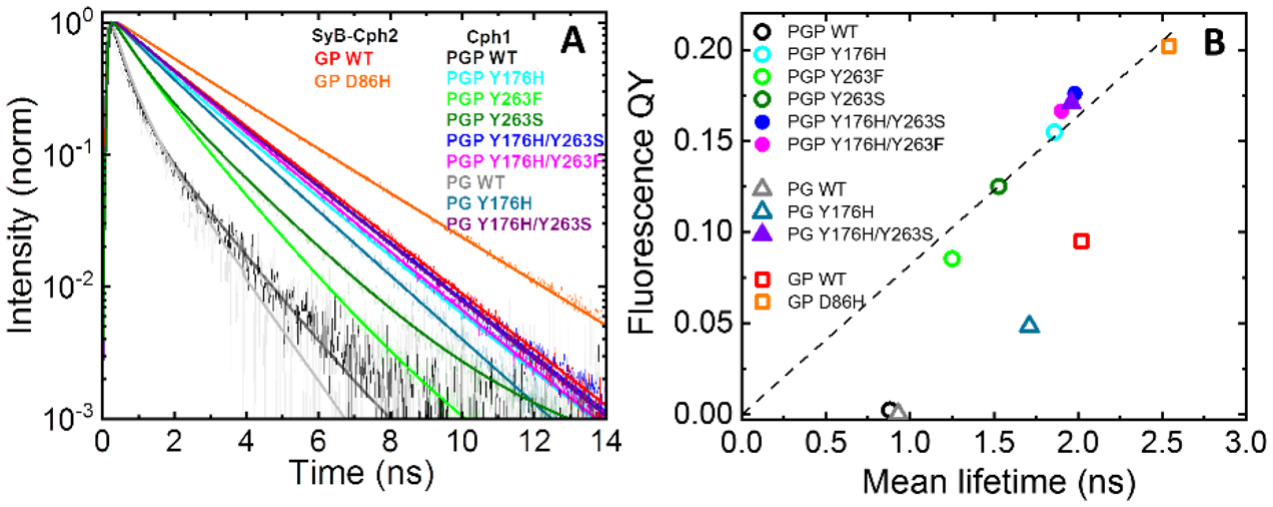
Fluorescence decay curves and correlation of fluorescence lifetimes with quantum yield. (**A**) Normalized fluorescence decay curves of Cph1 PGP, Cph1 PG, *SyB*-Cph2 GP and their variants. The unnormalized decay curves are shown in Fig. S6. The fit to a multi-exponential decay function (Eq. 2) is shown as a solid line. (**B**) Correlation between fluorescence quantum yield and mean fluorescence lifetime. The dotted line is to guide the eye. The slope of this line would correspond to the radiative rate *k*_r_ according to the equation ${\Phi}_{f}={k}_{r}\cdot \tau$ and indicate a similar *k*_*r*_ for those data points that fall on this line. Conditions: 300 mM NaCl, 50 mM Tris/HCl pH 7.8, 19 °C; λ_ex_ = 640 nm, and λ_em_ = 708 ± 38 nm.

To obtain further insight into the fluorescence properties and photophysical parameters of the new NIR-FP constructs, we measured the fluorescence lifetimes of Cph1 and Cph2 variants in the dark-adapted Pr state. Figure 4A and Fig. S6 show the fluorescence decay curves of Cph1 PGP, Cph1 PG, Cph2 GP, WT and variants. All samples were measured under the same conditions (see Materials and methods). The fluorescence decay curves were fitted with a biexponential model function. The fit results are summarised in Table S2. Mean fluorescence lifetimes and fluorescence quantum yield along with other summary parameters are shown in Table 1.

Cph1 PGP WT is weakly fluorescent in the Pr state, giving Φ_*f*_ and τ_mean_ values of 0.024 and 0.86 ns, respectively. Both values are significantly higher in the Y176H, Y263F and Y263S mutants. Our new double mutant NIR-FPs combining Y176H with Y263F or S show further increases, with Φ_*f*_ and τ_mean_ values of about 0.17 and 2 ns, respectively. The fluorescence lifetimes of the Cph1 PG WT and Y176H constructs in which the PHY domain is absent showed lower Φ_*f*_ and τ_mean_ values than their PGP homologs, whereas Y176H/Y263S in the PG background showed comparable fluorescence parameters to those in PGP. Finally, we characterised *SyB*-Cph2 GP WT and its D86H mutant. *SyB*-Cph2 WT as Pr gave Φ_*f*_ and τ_mean_ of about 0.12 and 2 ns, respectively—much larger values than for Cph1 WT. D86H in this background gave Φ_*f*_ and *τ*_*mean*_ values of about 0.2 and 2.6 ns.

As indicated above, the increases in fluorescence quantum yield and fluorescence lifetime are expected to be correlated. Indeed, most values for the single and double mutants are well correlated (dotted line to guide the eye in Fig. 4B). For those protein data points that fall on this line, the radiative rate *k*_r_ is quite similar and thereby implying a similar fluorescence mechanism. Strictly speaking, this relationship applies to samples with monoexponential fluorescence decays (see Table S2 and discussion below). Interestingly, the WTs of Cph1 PGP, Cph1 PG, and *SyB-*Cph2 show a different correlation and indicate lower radiative rates.

All our samples showed biexponential fluorescence decay rates (Table S2), suggesting either a heterogeneous population of fluorescent species in the ground state or an additional pathway for depopulation of the excited state (e.g. by quenching). However, we note that the amplitude of the longer lifetime component increases from around 20–30% in WT to about 90% in the Cph1 mutants (Table S2). This high population of the long fluorescence decay component is consistent with the reported single exponential fluorescence decays of fluorescent mutant phytochromes^46,50^.

The mean fluorescence lifetime of 1.9 ns reported here for Cph1 PGP Y176H agrees well with the single exponential lifetime of 1.8 ns published earlier for this mutant^50^. The even longer fluorescence decays of the new NIR-FP Cph1 double mutants described here are also almost monoexponential (~ 90% amplitude), indicating either more homogeneous populations or reduced access to alternative de-excitation processes in these mutants.

### Fluorescence imaging microscopy of *E.coli* cells

**Figure 5:**
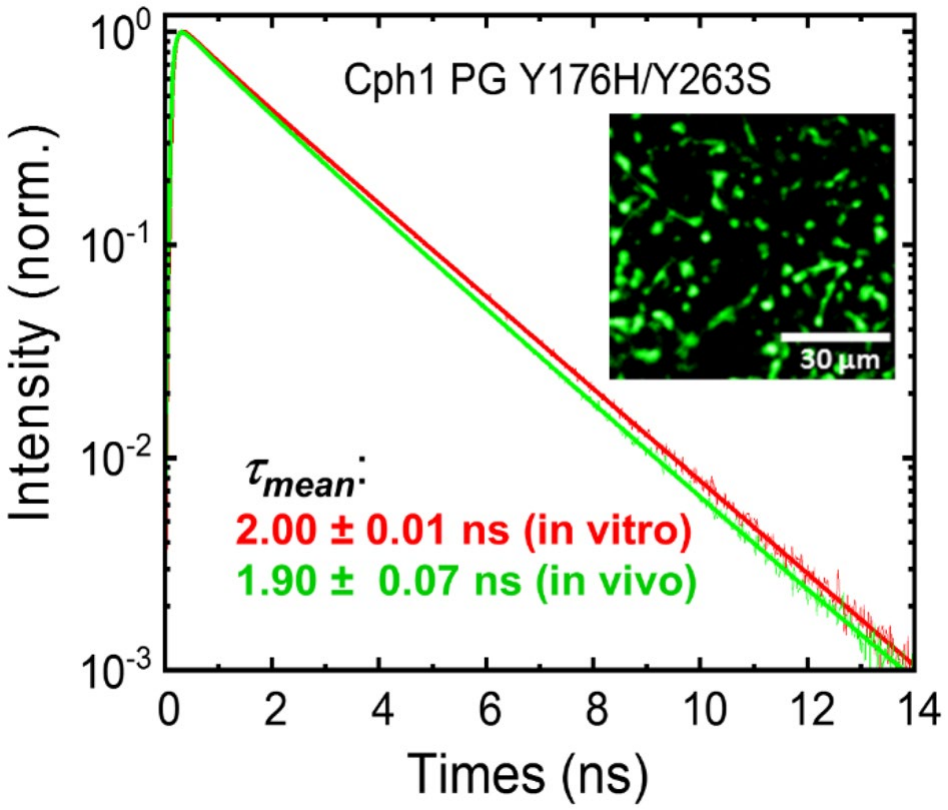
Characterization of Cph1 PG Y176H/Y263S in vivo using FLIM. Fluorescence decay curves in vitro and in vivo are shown together with the mean fluorescence lifetimes. The inset shows the FLIM image of *E. coli* cells expressing Cph1 PG Y176H/Y263S.

As a proof of concept for using the new NIR-FP variants as fluorescent tags for imaging, fluorescence lifetime imaging microscopy (FLIM) was carried out using *E. coli* cells expressing the appropriate genes^51^. Figure 5 shows the FLIM image of *E. coli* cells expressing Cph1 PG Y176H/Y263S (inset) and the corresponding fluorescence decay. For comparison, the fluorescence decay curve of the purified protein and the corresponding fluorescence lifetimes are indicated. Indeed, the values from *E.coli* and the purified protein of 1.9–2.0 ns are similar (Table S3). Thus, the long fluorescence lifetime of this mutant, and thereby the high fluorescence quantum yield, is translated into live cell imaging. As the fluorescence lifetime is directly correlated to the fluorescence quantum yield (see Fig. 4) and the extinction coefficient should be the same as under in vitro conditions, we assume a similar molecular brightness of the constructs in *E. coli* cells and in vitro. The effective brightness would only drop by a few percent compared to the molecular brightness when using the common laser line of 640 nm for excitation as the λ_max_ of the construct is 643 nm. The in vivo brightness, however, is determined not only by the molecular brightness or the effective brightness as determined by the used laser lines and filter setting, but other factors such as expression cell type and expression efficiency play a role^52^.

For future expression in mammalian cells it must be noted that unlike bacteriophytochromes, Cph1-based fluorophores cannot assemble with the biliverdin present in mammalian cells. However, the PCB cofactor can be fed to the cells^53^ or generated from the biliverdin by co-expressing pcyA (phycocyano-bilin:ferredoxin oxidoreductase) from the transfected plasmid^54,55^. Expressing HY2 (phytochromobilin:ferredoxin oxidoreductase) might be possible too^56^.

## Conclusions

In the present study we describe new, highly fluorescent Cph1 variants achieved by combining single mutations known to enhance fluorescence in various phytochromes. The effect of Y176H and Y263F/S appear synergistic, allowing Φ_*f*_ values of up to 0.17. Although removal of the PHY domain reduced fluorescence in the WT and Y176H, for unclear reasons it hardly affected the Y176H/Y263S mutant.

Viewed collectively, fluorescence studies of various mutants imply differences in the mechanisms of photoconversion between BV-utilising bacteriophytochromes and phytobilin-binding phytochromes (i.e., plant phytochromes and Cph1). Functions of even the highly conserved amino acids in the bilin pocket are not necessarily conserved. Mutations at several such loci that increase fluorescence quantum yield in one class of phytochromes do not always have the same effects in the other. Consequently, two classes of phytochromes must be regarded as different bases for the development of near-IR fluorophores. BV-binding bacteriophytochromes have been used intensively in this^57^, numerous rounds of mutagenesis having achieved Φ_*f*_ values of ~ 0.1. The present study, however, presents an alternative approach based on the phytobilin-binding Cph1 where mutations known to enhance fluorescence were combined, resulting in variants carrying only two mutations yet featuring very efficient fluorescence (Φ_*f*_ = 17% in the case of Cph1 PG Y176H/Y263S, Table S1). For further developments of Cph1-based fluorophores, we expect that additional, judiciously placed mutation(s) might enhance fluorescence still further.

## Materials and methods

### Site directed mutagenesis, protein expression and purification

Recombinant holophytochrome sensory module fragments (Cph1(PAS-GAF-PHY or PGP), residues 1–514; Cph1(PAS-GAF or PG), representing residues 1–322; *SyB*-Cph2(GAF-PHY or GP), residues 1–426; all with C-terminal H_6_ tags) were produced as PCB adducts in vivo by co-expressing hemeoxygenase and biliverdin-ferredoxin oxidoreductase alongside the apophytochrome in *E. coli*^51^ followed by lysis and purification as described^7,27^. Mutations were introduced into the coding sequences in the expression vector using either the Quikchange mutagenesis protocol (Agilent) or the back-to-back primer method. An alignment of the proteins used and key homologous proteins are provided in Fig. S7. For work under “safe light” conditions, LED headlamps with emission maximum at 490 nm were used.

### UV–vis, fluorescence, and CD spectroscopy

Absorption spectra were measured using an Agilent 8453 UV–vis diode array detector spectrophotometer modified to allow samples to be photoconverted by 655 or 730 nm LEDs in situ. *SyB*-Cph2 was photoconverted with a 600 nm LED. Chromophore configurations around the C15 = C16 double bond in holophytochromes following red or far-red irradiation were probed by denaturation in acidic urea as described^34,58^. This procedure also allowed us to estimate the extinction coefficient. We noted that these values from the in vivo assembly method were about 25% higher compared to the literature values based on the in vitro assembly reconstitution method of apoprotein. When used for comparison, the extinction coefficients were normalized to the published value of Cph1 Y176H^12^.

Fluorescence measurements were carried out using phytochromes in the Pr state following irradiation at 730 nm for 2 min. Excitation and emission spectra of the samples were measured using a Fluoromax4 spectrofluorometer (Horiba/Jobin Yvon) following essentially the same protocol as previously reported^27^ unless otherwise stated. Protein samples of approximately 0.1 mg/ml were used to minimise excitation light gradients and fluorescence self-absorption: higher concentrations were shown to lead to substantial artefacts in the measured spectra (see SI). Low intensity excitation light was employed to minimise photoconversion of samples. Emission spectra were obtained by exciting the sample at 610 nm using 0.3 nm slit bandwidth together with a 610 nm interference filter (T = 27.5%). Emission spectra 630–750 nm were measured with a 10 nm slit bandwidth and a 610 nm cut-off filter (T_630–750 nm_ = 94.2%) to exclude the scattering from the excitation light. Excitation spectra were obtained by measuring fluorescence at 720 nm with 10 nm slit bandwidth) as a function of excitation 580–700 nm (1 nm slit bandwidth). A grey filter (T_580-700 nm_ = 5.8%) and a 720 nm interference filter (T = 92.5%) were implemented in the optical path at the entrance and exit of the sample chamber, respectively. Fluorescence quantum yield Φ_f_ of the different samples were determined according to

1 $$\Phi _{f} = \frac{F}{{F^{{pheo}} }} \cdot \frac{{(1 - T)^{{pheo}} \cdot \Phi _{f}^{{pheo}} }}{{(1 - T)}},$$

using pheophytin A in diethyl ether at approximately 3 mM as the reference substance with fluorescence F^pheo^ and a ${\Phi }_{f}^{pheo}$ of 0.020^59,60^. F is the fluorescence and T is the transmittance of the sample.

CD spectra of samples were measured using Jasco J-715 as described^27^.

### Time-resolved fluorescence and fluorescence lifetime imaging microscopy

The holoprotein diluted to 4 µM with the standard buffer 50 mM Tris/HCl, 300 mM NaCl pH 7.8. 10 µl in Cellview dishes (Greiner Bio-One, Kremsmünster, Austria) was used for each measurement. The sample was irradiated with a far-red light emitting LED to achieve pure Pr. For FLIM of *E. coli* cells expressing PG Y176H/Y263S, the transformed *E. coli* cells were induced by 1 mM IPTG and 0.2% (w/v) L-arabinose overnight at 18 °C. The cell culture at OD_600 nm_ = 0.4 in PBS buffer was used for the measurement.

Time-resolved fluorescence measurements of Cph1 and Cph2 WT and variants were conducted in a home-built FLIM setup^61–64^ comprising an inverted Microscope (IX71, Olympus, Shinjuku, Japan), a tunable ps-supercontinuum laser (SuperK Extreme EXU-3, NKT Photonics, Blokken, Denmark), a confocal scanning unit (DCS120, B&H, Berlin, Germany), a hybrid PMT detector (HPM100-40, Becker & Hickl, Germany) and time-correlated single photon counting (TCSPC) electronics (SPC160, Becker & Hickl, Germany). The FLIM images were recorded using a 60 × objective (UPLSAPO60XW, Olympus) resulting in a total field of view with a side length of 300 µm. The proteins were excited at 640 nm using an acousto-optical tunable filter (SELECT UV–VIS, NKT Photonics) at 19.5 MHz. Fluorescence emission was spectrally selected by a long-pass filter LP 708 ± 38 nm (BrightLine HC, Semrock, Rochester, NY). Emitted photons were collected into 1024 time channels with a channel width of 19.97 ps. FLIM data were analysed using self-written routines in C^++^^31^. The recorded fluorescence decay traces were fitted using a multiexponential model function:

2 $$I\left(t\right)=\sum_{i}^{n}{\alpha }_{i}{e}^{-\frac{t}{{\tau }_{i}}}$$

with n the total number of decay components, ${\tau }_{i}$ the amplitude, and ${\alpha }_{i}$ the fluorescence lifetime of the ith component^30^. The mean fluorescence lifetime ${\tau }_{mean}$ was calculated by

3 $${\tau }_{mean}=\sum\limits_i^n {\frac{{{\alpha _i}{\tau _i}}}{{\sum\nolimits_i^n {{\alpha _i}{\tau _i}} }}} {\tau _i}$$

### Transient absorption flash photolysis setup

The transient changes of the dark-adapted Pr state of the Cph1 WT and variants after light activation were measured with a purpose-built flash photolysis spectrometer. The probes were excited using single ~ 3 ns flashes from a mid-band OPO (Horizon, Amplitude) pumbed by an Nd:YAG Laser (Surelite EX, Amplitude) at 640 nm with an pulse energy of 20 mJ. A photomultiplier tube (PMT, S4710, Hamamatsu) recorded changes in absorption at 705 nm of the excited sample. Possible scattered excitation laser light was suppressed by a 645 long pass filter (RG-645, Schott) and a monochromator. The changes in PMT voltage before excitation (U_0_) and over time (U(t)) were recorded using an oscilloscope (9350, LeCroy) and a data acquisition system (NI-USB-6009, National Instruments). Data were processed in a self-written LabView program to calculate the changes in absorption over time (ΔA(t)) as

4 $$\Delta A\left(t\right)=ln\frac{{U}_{0}}{U\left(t\right)}.$$

Single shot experiments are presented, and the transient is fitted by a sum of *n* exponentials with the time constants τ_n_ of the different intermediates and their respective amplitudes A_n_

5 $$\Delta A\left(t\right)= \sum_{n}{A}_{n}{{e}^{t/{\tau}_{n}}}.$$

## Supplementary Information

Supplementary Information.
