# Supplementary material for: Improved fluorescent phytochromes for in situ imaging
Source: Sci Rep. 2022 Apr 4;12:5587. doi: 10.1038/s41598-022-09169-x (PMC8980088; doi:10.1038/s41598-022-09169-x)
Supplement: Supplementary file 1 — Supplementary Information. [file 41598_2022_9169_MOESM1_ESM.pdf]

Supporting Information

Improved fluorescent phytochromes for *in situ* imaging

Soshichiro Nagano[1&], Maryam Sadeghi[2&], Jens Balke[2], Moritz Fleck[1], Nina Heckman[1], Georgios Psakis[1‡], Ulrike Alexiev[2*]

[1]Justus-Liebig-Universität, Institut für Pflanzenphysiologie, D-35390 Giessen, Germany

[2]Freie Universität Berlin, Institut für Experimentalphysik, D-14195 Berlin, Germany

[‡]Present address: University of Malta, Faculty of Health Sciences, Department of Food Sciences & Nutrition, Mater Dei Hospital, Msida, MSD 2080, Malta.

[&] These authors are equally contributed to this work.

[*]Corresponding author. E-mail address: ulrike.alexiev@fu-berlin.de

Contents

Figure S1: Configuration of the bilin in holo-phytochromes …………………………….......S3

Figure S2: CD spectra of Cph1 PGP WT and the Y176H mutant ……………………………S4

Figure S3: Flash photolysis data of Cph1 PGP WT and the mutant Y263S at 705 nm………..S5

Figure S4: Fluorescence emission spectra of Cph1 and SyB-Cph2 phytochromes…………...S6

Figure S5: Decrease in red/Soret peak ratio of excitation spectra as a function of protein concentration. ……………………………………………………………………………………..S7

Figure S6: Fluorescence lifetime curves (unnormalized)………………………………………S8

Figure S7: Aligned amino acid sequences of Cph1, SyB-Cph2 and other red fluorescent proteins………………………………………………………………………………………….S9

Table S1: Comparison of spectral characteristics of selected NIR-FPs………………………S10

Table S2: Fluorescence decay fit results………………………………………………………S11

Table S3: Fluorescence decay fit results of PG Y176H/Y263S *in vitro* and in *E. coli* cells……………………………………………………………………………………………S11

References…………………………………………………………………..……..……S12

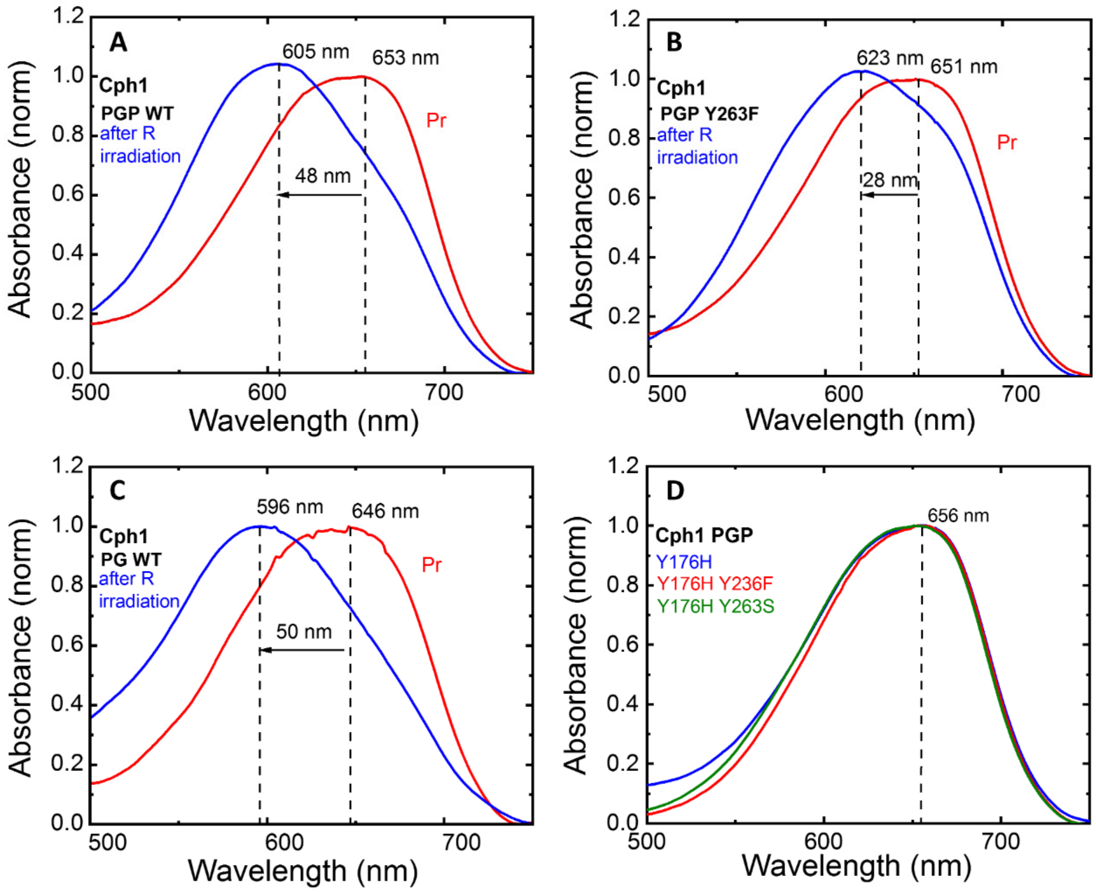

Figure S1: Configuration of the bilin in holo-phytochromes. Absorption spectra of phytochromes denatured by acid-urea were measured following irradiation by saturating red or far-red light (R or FR) [1]. Configuration of the bilin chromophore changes from 15Z to 15E upon R irradiation in Cph1 PGP WT (A), Cph1 PGP Y263F (B), Cph1 PG WT (C). The smaller shift in PGP Y263F than in the WT is explained by the lower proportion of Pfr formed. The chromophore of the Y176H, Y176H/Y263F and Y176H/Y263S mutants is in the *15Z* configuration (D), consistent with their Pr-like absorption spectra. After R irradiation followed by denaturation the spectra were virtually unchanged (data not shown), consistent with their minimal photochromicity in the native state.

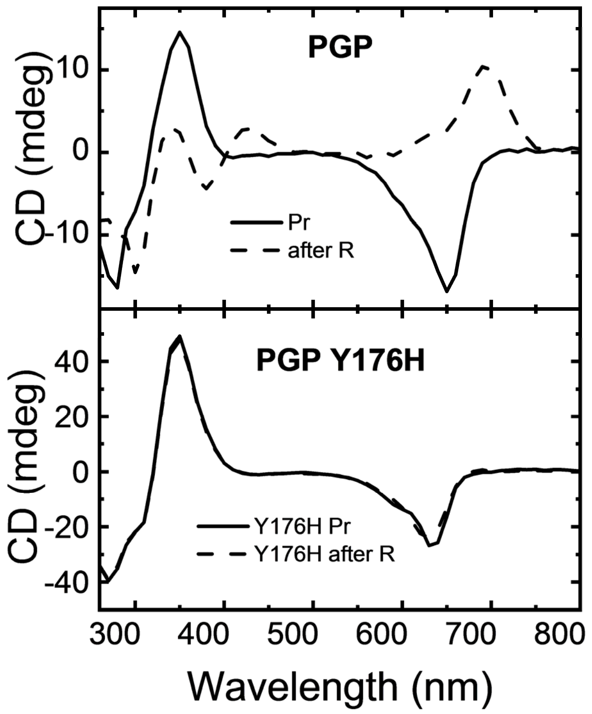

Figure S2: CD spectra of Cph1 PGP WT and the Y176H mutant. CD spectra of proteins as Pr and after R (645–665 nm) irradiation were measured using a Jasco J-715 spectrometer as described earlier [2]. In Cph1 PGP WT the photoconversion is associated with a change in CD signal from negative to positive in the red region, probably resulting from a change in *D*-ring disposition from α- to β-facial side of the PCB plane[3,4]. The equivalent change does not occur in Cph1 PGP Y176H, and the negative signal in the red region indicates the likely *D*-ring disposition on the α-facial side.

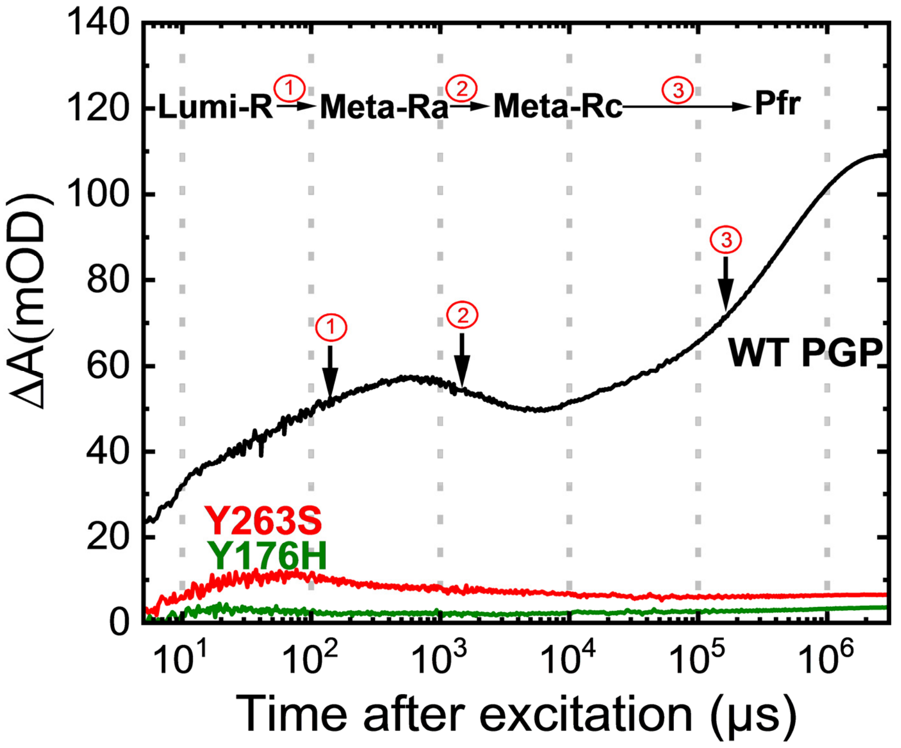

Figure S3: Flash photolysis data of Cph1 PGP WT, Y176H and Y263S at 705 nm. Cph1 PGP WT, Y176H, and Y263S absorbance difference transients are shown in black, green, and red, respectively. The samples were excited in the dark-adapted Pr state with 3 ns laser pulses at λ_{ex}= 640 nm. Transient absorption changes were measured at 705 nm. Sample conditions: 40 μM holoprotein in 150 mM NaCl, 50 mM Tris/HCl pH 7.8 at 20°C.

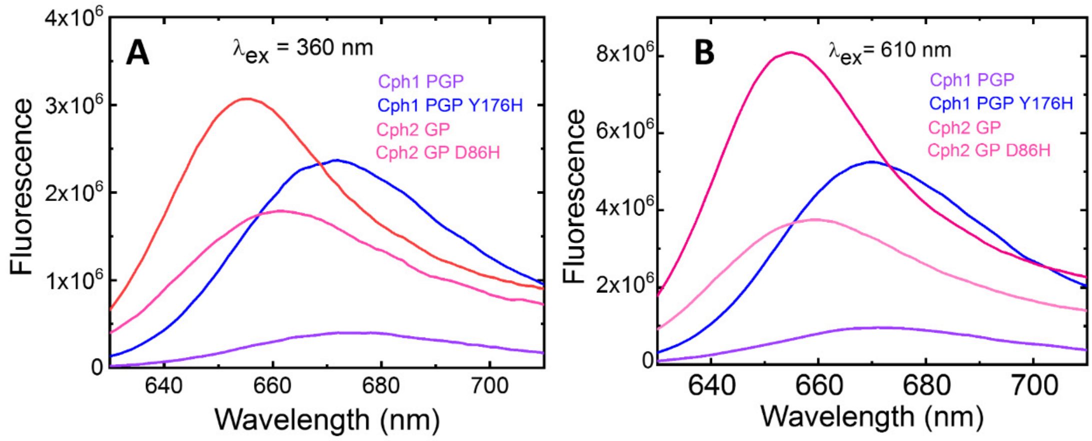

Figure S4: Fluorescence emission spectra of Cph1 and SyB-Cph2 phytochromes when excited at two different wavelengths. Fluorescence emission between 630-710 nm was measured by exciting samples (A) at 360 nm and (B) at 610 nm. No interference filter (610 nm) was used. Concentrations were approximately 0.1 mg/ml, and fluorescence values were adjusted by the absorbance of samples at corresponding excitation wavelengths. The entrance and exit slit bandwidths were 1 nm. The ratio of fluorescence (area under curve) of SyB-Cph2 D86H and Cph1 PGP Y176H was 1.26 and 1.49 when excited at 360 nm and 610 nm, respectively. These ratios are similar to the 1.38 value derived from fluorescence quantum yields determined independently in the present study (0.200 and 0.145, see **Table 1**), refuting the notion that SyB-Cph2 GP D86H is 5-fold more fluorescent than Cph1 PGP Y176H [5].

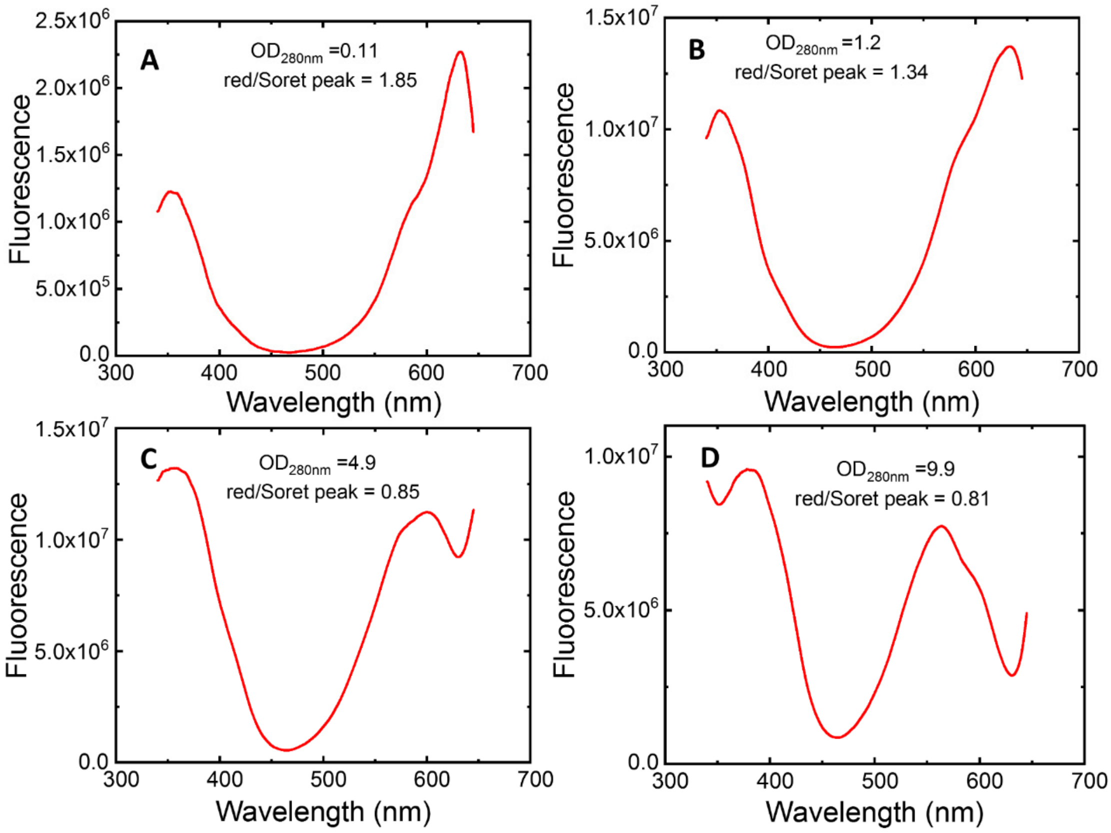

Figure S5: Decrease in red/Soret peak ratio of excitation spectra at different protein concentrations. A) Excitation spectrum at the lowest concentration is similar in shape to that of the absorbance spectrum (**Fig. 1** in main text). B-D) The red/Soret peak height ratio decreases with increasing concentration. At the highest concentration (D) the excitation spectrum resembles that of Fig. 6 (bottom panel) in Ulijasz *et al*., 2008[5]. This phenomenon can be explained by high absorbance in the red region of the sample. A protocol emulating that of Ulijasz *et al*. 2008[5] was employed here. Fluorescence of SyB-Cph2 GP D86H at the emission maximum wavelength as determined in the present study (655 nm) was measured following sample excitation between 340-645 nm. The entrance and exit slit bandwidths were 1 nm each. No optical filter was employed for these measurements. $A_{280\ nm}$ in (A) and (B) were measured with an UV–Vis spectrophotometer 8453 (1 cm pathlength; Agilent) and in (C) and (D) with a NanoVue Plus (GE Healthcare; 0.5 mm pathlength), both calculated for 1 cm pathlength.

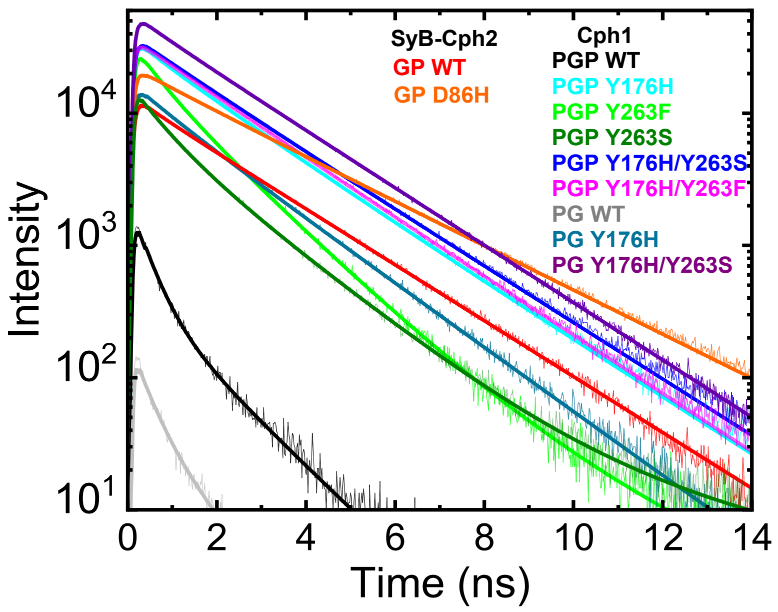

Figure S6: Fluorescence lifetime curves (unnormalized). Fluorescence decay curves of Cph1 PGP, Cph1 PG, SyB-Cph2 GP and their variants. Measurement time: 300s for each sample under identical excitation conditions (see Material and Methods). The fit to a multi-exponential decay function is shown as a solid line.

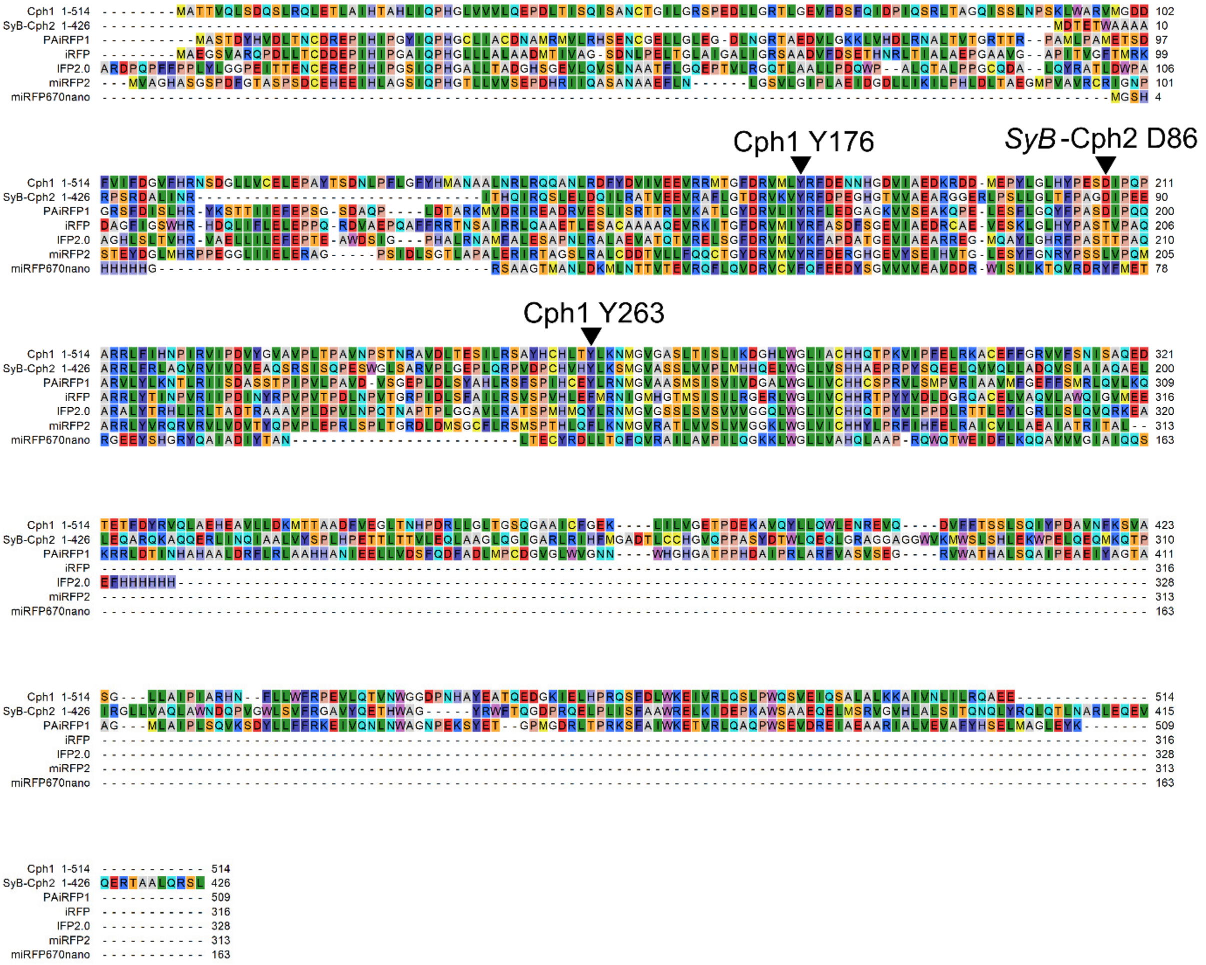

Figure S7: Aligned amino acid sequences of Cph1, SyB-Cph2 and other red fluorescent proteins. Key residues are indicated by triangles (Cph1 Y176 and Y263, SyB-Cph2 D86). The sequences were aligned using Clustal 2.1 using default settings and viewed using CLC sequence viewer.

Table S1: Comparison of spectral characteristics of selected NIR-FPs. The relative brightness is calculated by normalizing the molecular brightness values (fluorescence quantum yield x extinction coefficient) to 100% for Cph1 PGP Y176H with a molecular brightness of 10585 and an extinction coefficient of 73000 M^{-1} cm^{-1}.

Protein	$\lambda_{abs, max}$ (nm)[a]	$\lambda_{em, max}$ (nm)[b]	Φ_F [c]	Molecular Brightness relative to Y176H (%)	Chromophore	Reference
**Cph1 PGP Y176H**	642	672	0.145	100	PCB	Fischer and Lagarias, 2004, Fischer et al. 2005 [6]
**Cph1 PG Y176H Y263S**	642	670	0.176	143	PCB	This work
***SyB*-Cph2 D86H**	632	655	0.202	120	PCB	This work
**miRFP2**	676	706	0.043	23	BV	Babakhanova et al. 2021 [7]
**PAiRFP1**	690	717	0.048	30	BV	Piatkevich et al. 2013 [8]
**iRFP**	690	713	0.059	47	BV	Filonov et al. 2011 [9]
**iRFP670**	643	670	0.111	120	BV	Shcherbakova and Verkhusha, 2013 [10]
**IFP2.0**	690	711	0.080	65	BV	Yu et al. 2014 [11]
**miRFP670nano**	645	670	0,108	97	BV	Oliinyk et al. 2019 [12]
**smURFP** [d]	642	670	0.180	306	BV	Rodriguez et al. 2016 [13]

[a] $\lambda_{abs, max}$ –absorbance peak; [b] $\lambda_{em, max}$ –fluorescence emission peak; [c] Φ_F – fluorescence quantum yield; [d] for comparison a phycobiliprotein-based construct (smURFP) is listed.

Table S2: Fluorescence decay fit results. The lifetime components τ_1, τ_2 and the corresponding relative amplitudes α_1 and α_2 from the fit are given. Errors were estimated by the exhaustive error anlysis method using the software Globals (Laboratory for Fluorescence Dynamics, University of California, Irvine, CA). The reduced chi-square (χ_{red}^2) is given to judge the goodness of the fit.

Construct	τ_1 (ns)	τ_2 (ns)	α_1 (%)	α_2 (%)	χ_{red}^2
PGP WT	0.32 ± 0.01	1.25 ± 0.02	76.09 ± 1.36	23.91 ± 0.82	1.09
PGP Y176H	0.90 ± 0.05	2.00 ± 0.01	18.02 ± 0.90	81.98 ± 0.90	1.27
PGP Y263F	0.68 ± 0.01	1.47 ± 0.01	43.26 ± 0.56	56.74 ± 0.84	1.14
PGP Y263S	0.69 ± 0.03	1.76 ± 0.01	50.30 ± 0.90	49.70 ± 0.90	1.19
PGP Y176H Y263S	0.84 ± 0.08	2.064 ± 0.008	10.14 ± 0.97	89.86 ± 0.97	1.14
PGP Y176H Y263F	0.42 ± 0.03	1.955 ± 0.002	8.64 ± 0.45	91.36 ± 0.45	1.23
PG WT	0.19 ± 0.20	0.86 ± 0.02	61.32 ± 1.74	38.68 ± 2.32	0.97
PG Y176H	0.70 ± 0.04	1.817 ± 0.006	15.23 ± 0.41	84.77 ± 0.82	1.11
PG Y176H Y263S	0.76 ± 0.05	2.050 ± 0.004	8.70 ± 0.48	91.30 ± 0.97	1.32
GP WT	0.60 ± 0.05	2.120 ± 0.005	9.36 ± 0.49	90.64 ± 0.50	1.11
GP D86H	0.91 ± 0.01	2.65 ± 0.01	7.50 ± 0.62	92.50 ± 3.12	1.23

Table S3: Fluorescence decay fit results of Cph1 PG Y176H/Y263S *in vitro* and in *E. coli* cells. The lifetime components τ_1, τ_2 and the corresponding relative amplitudes α_1 and α_2 from the fit are given. Errors were estimated by the exhaustive error anlysis method using the software Globals (Laboratory for Fluorescence Dynamics, University of California, Irvine, CA). The reduced chi-square (χ_{red}^2) is given to judge the goodness of the fit.

Construct	τ_1 (ns)	τ_2 (ns)	α_1 (%)	α_2 (%)	τ_{mean} (ns)	χ_{red}^2
PG Y176H/Y263S (*in vitro*)	0.76 ± 0.05	2.05 ± 0.01	8.70 ± 0.48	91.30 ± 0.48	2.00 ± 0.01	1.32
PG Y176H/Y263S (in *E. coli*)	1.17 ± 0.04	2.03 ± 0.01	22.48 ± 1. 83	77.52 ± 1. 83	1.90 ± 0.07	1.51

References

1 Song, C. *et al.* The D-ring, Not the A-ring, Rotates in Synechococcus OS-B′ Phytochrome*. *Journal of Biological Chemistry* **289**, 2552-2562, doi:10.1074/jbc.M113.520031 (2014).

2 Mailliet, J. *et al.* Spectroscopy and a High-Resolution Crystal Structure of Tyr263 Mutants of Cyanobacterial Phytochrome Cph1. *Journal of Molecular Biology* **413**, 115-127, doi:10.1016/j.jmb.2011.08.023 (2011).

3 Rockwell, N. C., Shang, L., Martin, S. S. & Lagarias, J. C. Distinct classes of red/far-red photochemistry within the phytochrome superfamily. *Proceedings of the National Academy of Sciences* **106**, 6123-6127, doi:10.1073/pnas.0902370106 (2009).

4 Song, C. *et al.* Two ground state isoforms and a chromophore D-ring photoflip triggering extensive intramolecular changes in a canonical phytochrome. *Proceedings of the National Academy of Sciences of the United States of America* **108**, 3842-3847, doi:10.1073/pnas.1013377108 (2011).

5 Ulijasz, A. T. *et al.* Characterization of two thermostable cyanobacterial phytochromes reveals global movements in the chromophore-binding domain during photoconversion. *J Biol Chem* **283**, 21251-21266, doi:10.1074/jbc.M801592200 (2008).

6 Fischer, A. J. *et al.* Multiple Roles of a Conserved GAF Domain Tyrosine Residue in Cyanobacterial and Plant Phytochromes. *Biochemistry* **44**, 15203-15215, doi:10.1021/bi051633z (2005).

7 Babakhanova, S. *et al.* Rapid Directed Molecular Evolution of Fluorescent Proteins in Mammalian Cells. *Protein Science*, doi:10.1002/pro.4261 (2021).

8 Piatkevich, K. D., Subach, F. V. & Verkhusha, V. V. Far-red light photoactivatable near-infrared fluorescent proteins engineered from a bacterial phytochrome. *Nat. Commun.* **4**, 2153, doi:10.1038/ncomms3153 (2013).

9 Filonov, G. S. *et al.* Bright and stable near-infrared fluorescent protein for in vivo imaging. *Nat Biotechnol* **29**, 757-761, doi:10.1038/nbt.1918 (2011).

10 Shcherbakova, D. M. & Verkhusha, V. V. Near-infrared fluorescent proteins for multicolor in vivo imaging. *Nature Methods* **10**, 751-754, doi:10.1038/nmeth.2521 (2013).

11 Yu, D. *et al.* An improved monomeric infrared fluorescent protein for neuronal and tumour brain imaging. *Nat. Commun.* **5**, 3626, doi:10.1038/ncomms4626 (2014).

12 Oliinyk, O. S., Shemetov, A. A., Pletnev, S., Shcherbakova, D. M. & Verkhusha, V. V. Smallest near-infrared fluorescent protein evolved from cyanobacteriochrome as versatile tag for spectral multiplexing. *Nat. Commun.* **10**, 279, doi:10.1038/s41467-018-08050-8 (2019).

13 Rodriguez, E. A. *et al.* A far-red fluorescent protein evolved from a cyanobacterial phycobiliprotein. *Nature Methods* **13**, 763-769, doi:10.1038/nmeth.3935 (2016).
